# Forecasting Northward Range Expansion of Switchgrass in China via Multi-Scenario MaxEnt Simulations

**DOI:** 10.3390/biology14081061

**Published:** 2025-08-15

**Authors:** Yangzhou Xiang, Suhang Li, Qiong Yang, Jun Ren, Ying Liu, Yang Luo, Ling Zhao, Xuqiang Luo, Bin Yao, Xinzhao Guo

**Affiliations:** 1 School of Geography and Resources, Guizhou Education University, Guiyang 550018, China; 2 School of Biological Sciences, Guizhou Education University, Guiyang 550018, China; 3 State Power Investment Corporation Power Station Operation Technology (Beijing) Co., Ltd., Beijing 100032, China; 4 Institute of Ecological Conservation and Restoration, Chinese Academy of Forestry, Beijing 100091, China; 5 Guizhou Institute of Forest Inventory and Planning, Guiyang 550003, China

**Keywords:** switchgrass, species distribution modeling, parameter optimization, climate change, bioenergy crop

## Abstract

Global warming is accelerating the poleward and upward shifts in climatically suitable ranges of species. *Panicum virgatum* (switchgrass) is recognized for its dual value in China’s dual-carbon strategy: mitigating food–energy land competition and restoring marginal ecosystems. However, the accuracy of habitat projections is constrained by three limitations: reliance on North American provenance data, uncalibrated model parameters, and insufficient scenario coverage. To address these, 48 switchgrass occurrence records and 22 climatic–topographic variables were integrated. The MaxEnt model was optimized with ENMeval (RM = 4.0, FC = LQH) and coupled with three SSP scenarios (SSP1-2.6, SSP3-7.0, SSP5-8.5) to quantify habitat area changes and centroid shifts across China. The key findings were as follows: (1) The mean temperature of the coldest quarter (Bio11) and elevation were identified as the key limiting factors for the suitable distribution of switchgrass, with their corresponding optimal thresholds determined as −8.79 to 8.11 °C and 0 to 2893 m, respectively. (2) The current suitable habitat covers 583.58 × 10^4^ km^2^, concentrated in the North China Plain. (3) Under SSP5-8.5, the high-suitability habitat is projected to reach 229.44 × 10^4^ km^2^ by the 2090s, with the centroid migrating 305 km northwestward to the Inner Mongolia–Jilin belt. This study highlights the climate–topography coupling that drives northward migration and proposes cold-tolerant cultivar development, priority zoning of marginal lands, and ecological corridor establishment to inform climate-smart biomass energy planning in China.

## Simple Summary

*Panicum virgatum* (switchgrass), a key bioenergy crop to achieve China’s renewable goals, faces an uncertain future distribution under climate change. This study mapped its current suitable habitats and projected future changes through to 2090. Currently, 60.8% of China’s territory provides suitable conditions, concentrated in the North China Plain. Climate warming will significantly expand suitable areas northward (up to a +138% gain) but cause losses in the overheated southwest. Key constraints are the mean temperature of the coldest quarter (optimal: −8.8 °C to 8.1 °C) and elevation (optimal: 0–2893 m). Future strategies must focus on developing cold-tolerant varieties, utilizing marginal lands in newly suitable northern areas, and creating migration pathways. These insights enable climate-resilient bioenergy planning, supporting carbon neutrality without compromising food security.

## 1. Introduction

Global warming is reshaping terrestrial ecosystem patterns at an unprecedented rate and driving species distributions towards migration [1,2], which has been established as a critical issue at the intersection of ecology and agricultural science [3,4]. Within this context, *Panicum virgatum*, commonly known as switchgrass, is a perennial warm-season bunchgrass native to North America, where it occurs naturally from 55° N latitude in Canada southwards into the United States and Mexico. This species is internationally recognized as a key species underpinning the biomass energy transition, owing to its high photosynthetic efficiency and pronounced tolerance to poor soils [5,6]. Notably for China, switchgrass is ascribed dual value within the nation’s dual-carbon strategy: firstly, as a substitute for corn ethanol to alleviate the food-versus-fuel land competition, and secondly, by enabling ecological restoration and enhanced carbon sequestration through utilization of marginal lands [7,8]. However, the complex topographic tiers and pronounced climatic gradients characteristic of China [9,10] result in a highly fragmented pattern of suitable habitats for switchgrass. More critically, a temperature increase of up to 5 °C in northern China by the end of the 21st century is projected by Zhang et al. [11] under the SSP5-8.5 scenario, which is expected to fundamentally alter heat–precipitation coupling relationships. Consequently, investigating how climate change shapes the habitat dynamics of switchgrass is deemed essential for elucidating its biogeographic distribution patterns and informing energy security-related decision-making in China. Ecological niche-based distribution modeling employs statistical algorithms to quantify relationships between observed species occurrences and key environmental predictors, thereby characterizing habitat suitability patterns [12].

Globally, field-validated yield records for switchgrass converge on a robust range of 7–39 Mg DM ha^−1^ [13,14,15]. Zhang et al. [16] reported county-scale mean peak yields of 20 Mg ha^−1^ for lowland cultivars under rain-fed management, while Happs et al. [13] demonstrated that 84 natural variants achieve ≥ 7.5 Mg ha^−1^ in multi-site common gardens, with the top quartile reaching 39 Mg ha^−1^. These figures align with Zhao et al. [14], who measured sustained yields of 7–9 Mg ha^−1^ yr^−1^ over seven years on unfertilized marginal cropland in northern China, confirming the viability of low-input cultivation. Current utilization pathways are operational: over 60% of harvested biomass is delivered directly to local power plants via bale-and-chip logistics, with the remainder supplying pilot ethanol and fiberboard facilities [14]. Notably, southern lowland cultivars like Alamo yield 17.8 Mg ha^−1^ yr^−1^ under hotter, drier conditions [15], highlighting the importance of cultivar matching to the northward expansion zones projected by our model. Economically, Zhang et al. [16] calculated a net present value of USD 162–274 ha^−1^ yr^−1^, outperforming miscanthus (USD 152 ha^−1^ yr^−1^) on comparable land. Integrated assessments reveal that at yields above 20 Mg ha^−1^, ethanol production costs decrease to USD 0.64 L^−1^, making it competitive with maize ethanol while achieving a lifecycle carbon abatement of 517 g CO_2_e L^−1^ [13]. Collectively, these data position switchgrass as a crop capable of delivering high biomass yields, cost-effective bioenergy, and significant carbon mitigation across China’s emerging northern production belt, justifying the scenario analyses that follow.

Despite its introduction to China over three decades ago [17], the large-scale deployment of switchgrass remains constrained by two core limitations: spatial mismatch between current cultivation zones and areas of climatic suitability, and inadequate understanding of the dynamic evolution of suitable habitats under future climate scenarios. The former is manifested through unsystematic introduction planning—sustainability concerns have been raised in the North China Plain due to competition with staple food crops for arable land [18], while cultivation efficiency in the karst mountain regions of Southwest China has been significantly lower than anticipated due to insufficient climatic adaptability [19]. The latter limitation stems from a lack of quantitative projections regarding future climate responses, particularly the failure to delineate migration pathways of suitable habitats and their driving mechanisms. These shortcomings are rooted in three fundamental research gaps: (1) insufficient data representativeness, whereby reliance on North American distribution data or predictive conclusions [20] has resulted in misjudgment of suitable habitat ranges due to inadequate consideration of environmental tolerance thresholds (climate and topography) in locally adapted Chinese cultivars [21]; (2) fixed model parameters, where the widespread use of MaxEnt with uncalibrated default settings (e.g., RM = 1.0, FC = LQHP) has significantly elevated overfitting risks, consequently overestimating potential suitability in high-latitude regions while underestimating sensitivity to key seasonal temperature factors [22]; and (3) inadequate scenario coverage, as existing analyses are predominantly confined to single climate pathways [23], without integrating the synergistic effects of China’s renewable energy policies and diversified Shared Socioeconomic Pathways (SSPs) [24,25,26] and lacking quantitative assessments based on centroid migration trajectories and area gains/losses of suitable habitats to scientifically prioritize cross-regional germplasm resource allocation. Collectively, these deficiencies hinder the scientific planning of China’s “climate-smart” biomass energy corridors.

Our study aims to investigate how climate change will impact the distribution of switchgrass in China, a critical bioenergy crop. Specifically, we hypothesize that climate change will drive significant northward expansion of suitable habitats for switchgrass, altering its current distribution pattern. To test this hypothesis, we employed the MaxEnt model optimized with ENMeval and coupled it with three Shared Socioeconomic Pathway (SSP) scenarios (SSP1-2.6, SSP3-7.0, and SSP5-8.5) to project habitat changes from the 2050s to the 2090s. Our primary objectives are to (1) map the current suitable habitats of switchgrass in China, (2) quantify future changes in habitat area and centroid shifts under different climate scenarios, and (3) identify key environmental factors influencing its distribution. By achieving these objectives, we aim to provide evidence-based strategies for sustainable bioenergy planning under climate change, supporting China’s dual-carbon goals without compromising food security.

## 2. Materials and Methods

### 2.1. Switchgrass Occurrence Data Acquisition and Pre-Processing

**Figure 1:**
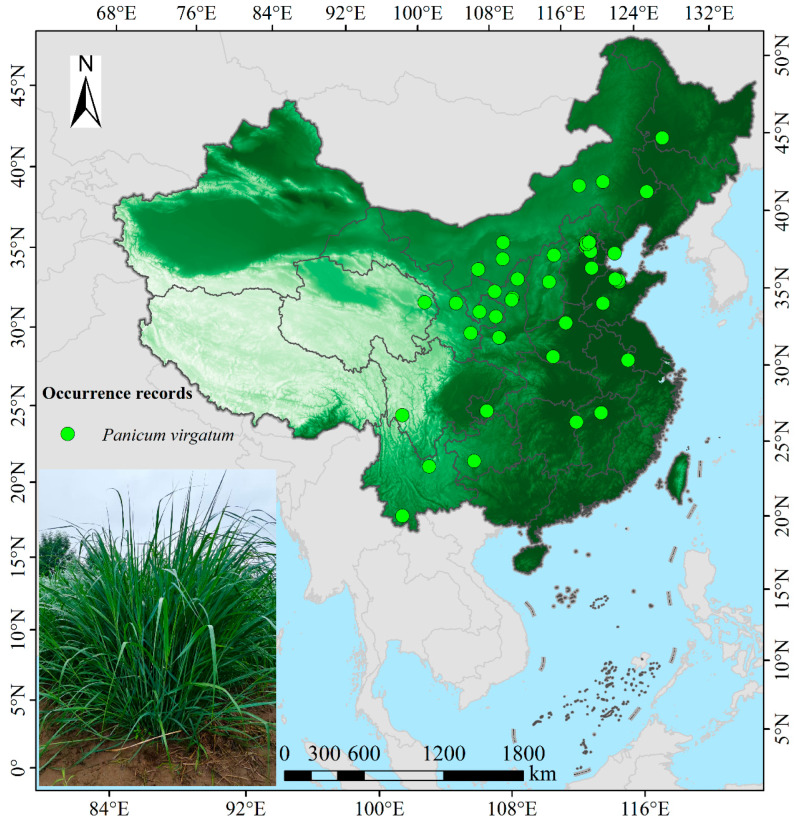
The occurrence records of switchgrass in China.

Geographic distribution data for switchgrass in China were primarily integrated from the following electronic repositories and academic platforms: Chinese Virtual Herbarium (https://www.cvh.ac.cn/, accessed on 12 March 2025), Global Biodiversity Information Facility (https://doi.org/10.15468/dl.4h3u35, accessed on 15 March 2025), National Specimen Information Infrastructure (http://www.nsii.org.cn/2017/home.php, accessed on 16 March 2025), as well as academic literature retrieved from China National Knowledge Infrastructure (https://www.cnki.net/, accessed on 18 March 2025), Web of Science (https://www.webofscience.com/, accessed on 18 March 2025), and Google Scholar (https://scholar.google.com, accessed on 18 March 2025). To eliminate spatial autocorrelation and enhance data reliability, a 2.5′ resolution grid was constructed in ArcGIS 10.8, whereafter all distribution records were projected into a unified coordinate system, and Euclidean distances from each point to its corresponding grid center were automatically calculated using Near Analysis. Subsequently, only the point nearest to each grid center possessing complete habitat information was retained, while the remaining records were excluded. Ultimately, 48 spatially independent occurrence points were identified (Figure 1), thereby providing robust data support for precision habitat suitability modeling. Future refinements should integrate soil stressors (texture and salinity) and extreme climate frequency to improve precision. Furthermore, the 1:10,000,000 administrative basemap of China was obtained from the standard map service website of the Ministry of Natural Resources (http://bzdt.ch.mnr.gov.cn, accessed on 22 December 2023) with the approval number GS (2023)2762. The original JPG image was georeferenced in ArcGIS 10.8 and subsequently exported as a Shapefile vector file within the GCS_WGS_1984 geographic coordinate system.

### 2.2. Environmental Data Collection and Processing

**Figure 2:**
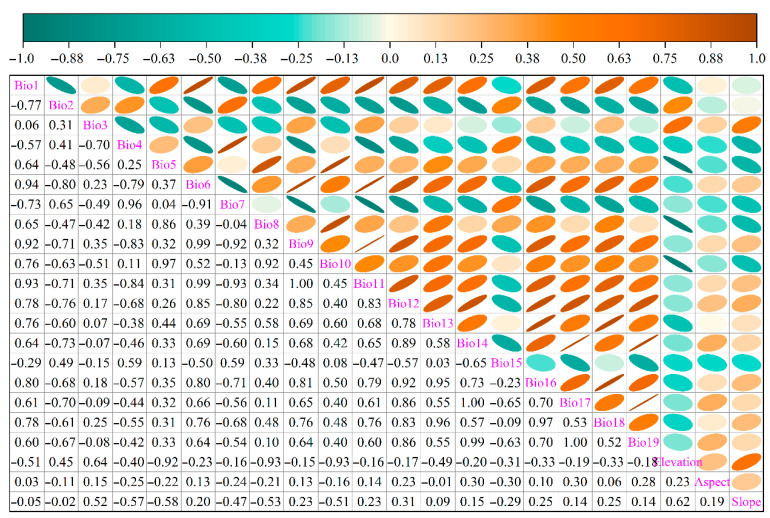
Spearman rank correlation matrix of the 22 environmental predictors considered in this study.

A total of 22 environmental predictors at 2.5′ × 2.5′ spatial resolution were compiled (Table 1), comprising 19 bioclimatic variables (bio1–bio19) and three topographic metrics (elevation, slope, and aspect). Bioclimatic variables for both current and future periods, together with elevation, were retrieved from WorldClim 2.1 (https://www.worldclim.org, accessed on 16 June 2023), whereas slope and aspect were derived from elevation via the Spatial Analyst “Slope” and “Aspect” tools in ArcGIS 10.8. To capture phased climate impacts on switchgrass distribution, future projections were generated with the CMIP6 model BCC-CSM2-MR, recognized as exhibiting the highest simulation accuracy over China [27], and were extracted for the 2050s (2041–2060), 2070s (2061–2080), and 2090s (2081–2100). Scenarios followed the IPCC AR6 Shared Socioeconomic Pathways (SSPs), specifically SSP1-2.6 (low forcing, ~2.6 W m^−2^ by 2100), SSP3-7.0 (medium–high forcing, ~7.0 W m^−2^), and SSP5-8.5 (high forcing, ~8.5 W m^−2^) [28]. Multicollinearity was mitigated by first conducting Spearman correlation tests on all 22 variables in Origin 2025b (Figure 2) and then integrating the contribution rates (Table 1) from MaxEnt 3.4.4 (http://biodiversityinformatics.amnh.org/open_source/maxent/, accessed 16 May 2025). When the correlation coefficient between two variables exceeded 0.75, the variable with the lower contribution was discarded. Consequently, eight non-redundant predictors were retained: isothermality (Bio3), mean temperature of coldest quarter (Bio11), precipitation of wettest month (Bio13), variation of precipitation seasonality (Bio15), precipitation of coldest quarter (Bio19), elevation, aspect, and slope.

### 2.3. MaxEnt Model Calibration and Configuration

#### 2.3.1. Model Calibration and Tuning

Potential distribution probabilities predicted by MaxEnt models employing default parameters may deviate from actual species distributions [29,30]. Consequently, the direct application of such models to predict switchgrass distributions in China may result in overestimation or underestimation of suitable habitat ranges [31]. Therefore, accurate assessment of climate change impacts on the future distribution of switchgrass necessitates optimization of MaxEnt model parameters. To achieve this, the ENMeval 2.0.4 package [32] within R 4.2.1 software was utilized for model calibration. Two core parameters were specifically targeted for adjustment: the regularization multiplier (RM) and feature combination (FC). Five fundamental feature types were incorporated within MaxEnt: linear (L), quadratic (Q), product (P), threshold (T), and hinge (H). Eight RM values (ranging from 0.5 to 4.0 in 0.5 increments) and nine FC combinations (L, H, LQ, LQH, LQHP, LQHPT, QHP, QHPT, and HPT) were tested. To evaluate model fit and complexity across different parameter combinations, Delta.AICc, OR10, and AUC.diff were selected as evaluation metrics [33]. The optimal parameter combination was subsequently identified and used to construct the final model.

#### 2.3.2. Parameter Settings

The parameter-optimized MaxEnt model (regularization multiplier (RM) = 4.0, feature combination (FC) = LQH) was employed to predict the potential suitable habitats for switchgrass. To ensure model accuracy and reliability, a random sampling approach was adopted. The species occurrence data for switchgrass were divided into two subsets: 75% of the data were utilized as the training dataset, while the remaining 25% constituted an independent testing dataset. Ten-fold cross-validation was implemented to enhance model robustness and control overfitting. The maximum number of background points was constrained to 10,000. The model ultimately output probability distribution in logistic format (.asc files). These outputs share identical geographic coordinate systems and spatial resolutions with the environmental predictor layers, enabling direct support for subsequent spatial pattern analysis and introduction priority assessment.

#### 2.3.3. Model Performance Assessment

Model performance in this study was quantified using the receiver operating characteristic (ROC) curve and its area under the curve (AUC) as core metrics. A systematic evaluation framework was constructed following the assessment protocol proposed by Phillips et al. [34]. The AUC value ranges between 0 and 1 [35,36], with higher values indicating greater predictive accuracy [37,38]. Predictive accuracy in MaxEnt modeling is conventionally classified into five levels based on AUC values [39]: (1) 0.50–0.60: model performance is considered statistically indistinguishable from random prediction (*P* > 0.05) and is therefore deemed ineffective; (2) 0.60–0.70: the model is considered to possess only fundamental discriminatory ability, and its reliability is assessed as low; (3) 0.70–0.80: model predictions are considered effective in discriminating suitable habitats and are confirmed to meet the basic requirements for ecological niche modeling; (4) 0.80–0.90: the model is regarded as having high prediction accuracy, and its spatial fit is judged to be significant; and (5) 0.90–1.00: model predictive capability is classified as excellent, and the actual species distribution pattern is considered to be highly restored.

### 2.4. Suitability Classification for Switchgrass

The averaged outputs from ten replicate MaxEnt model cross-validations (.asc format) were utilized as the foundation. These outputs were converted into .tif format (2.5 arc-minute resolution) using ArcGIS 10.8. Standardization procedures were applied to ensure spatial analysis compatibility and cartographic accuracy. Cluster analysis was performed based on the actual distribution frequency of switchgrass occurrences. The habitat suitability probability (*P*) was classified into four gradients using the Jenks natural breaks classification method: (1) non-suitable habitats (0 ≤ *P* < 0.1), characterized by significant suppression of population establishment due to environmental limiting factors; (2) low-suitability habitats (0.1 ≤ *P* < 0.3), reflecting marginal survival conditions under localized habitat stress; (3) medium-suitability habitats (0.3 ≤ *P* < 0.5), indicating suboptimal environments supporting stable population maintenance; and (4) high-suitability habitats (0.5 ≤ *P* ≤ 1.0), corresponding to core areas possessing optimal hydrothermal conditions and resource availability, where P represents the suitability index.

### 2.5. Dynamic Changes in Potential Suitable Habitats for Switchgrass

Dynamic changes in switchgrass suitable habitats were intensively analyzed in this study. Predictive models spanning multiple time periods were constructed to delineate habitat evolution into three categories: retained, lost, and gained areas. Specifically, areas with a habitat suitability probability (*P*) ≥ 10% were classified as suitable habitats (encompassing low-, medium-, and high-suitability areas), while areas with P < 10% were designated as non-suitable habitats. A presence/absence (1/0) matrix was generated for both current and future climate scenarios. Using the current extent of switchgrass suitable habitats as the baseline, future distributional area changes were quantified. Matrix value transitions carry explicit ecological interpretations: 0→1 denotes habitat gain (expansion), 1→0 indicates habitat loss (contraction), and 1→1 represents habitat retention (stability).

### 2.6. Centroid Shift of Suitable Habitats for Switchgrass

To analyze the centroid migration trends of switchgrass suitable habitats, the averaged probability outputs from ten MaxEnt model replicates were utilized as the base data. First, the probability raster was reclassified into a binary (0/1) format using the “Reclassify” tool in ArcGIS 10.8 software. Subsequently, suitable pixels were converted into vector point features using the “Raster to Point” tool. Weighted centroids at consecutive 0.1 probability thresholds were calculated using the “Mean Center” tool. The geographic coordinates of these centroids were extracted for the current period and for future periods (2050s, 2070s, and 2090s) under SSP1-2.6, SSP3-7.0, and SSP5-8.5 climate scenarios. Centroid data across multiple climate scenarios were integrated using the “Point Merge” tool. Finally, centroid migration trajectories were constructed using the “Points to Line” tool.

## 3. Results

### 3.1. Optimized Model Accuracy Assessment

Parameter optimization of the MaxEnt model was evaluated using Delta.AICc, OR10, and AUC.diff metrics. The default parameter set (FC = LQHP, RM = 1.0) produced a high Delta.AICc value of 31.229 (Table 2). In contrast, optimization employing FC = LQH and RM = 4.0 achieved a Delta.AICc of 0. Furthermore, significantly lower OR10 and AUC.diff values were observed for the optimized model compared to the default configuration, representing reductions of approximately 16.67% and 8.70%, respectively. This collective evidence indicates that parameter optimization substantially mitigated model overfitting. Thus, the configuration FC = LQH with RM = 4.0 was established as the optimal parameter set in this analysis.

**Figure 3:**
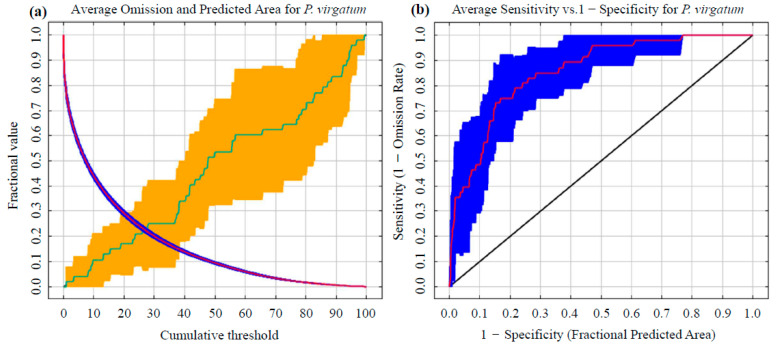
Performance assessment of the MaxEnt model predicting switchgrass distribution: (**a**) omission error rate and (**b**) receiver operating characteristic (ROC) curve.

Ten replicate MaxEnt (version 3.4.4) simulations were executed using the optimal parameter configuration (FC = LQH, RM = 4). These simulations were based on 48 spatially recorded switchgrass occurrence points and nine environmental variables (Bio3, Bio11, Bio13, Bio15, Bio19, elevation, aspect, and slope) previously screened for multicollinearity. The receiver operating characteristic (ROC) curve generated for the current scenario (Figure 3) yielded a training dataset AUC value of 0.856 ± 0.069, thereby demonstrating robust predictive accuracy of the optimized parameter set in capturing the climatic niche of switchgrass.

### 3.2. Key Environmental Drivers of Switchgrass Distribution

**Figure 4:**
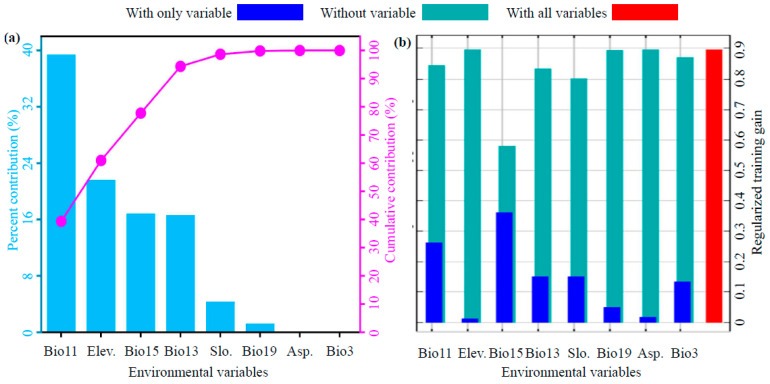
MaxEnt-derived relative contribution (**a**) and jackknife test of regularized training gain (**b**) for the eight environmental variables influencing switchgrass distribution.

Eight selected environmental factors were identified via analysis of the optimized MaxEnt model results in our study (Figure 4a). Their percent contributions to switchgrass distribution were quantified as follows: The mean temperature of coldest quarter (Bio11, 39.4%) was determined to be the primary driver; elevation (21.6%) was identified as the secondary driver, followed by variation of precipitation seasonality (Bio15, 16.8%), precipitation of wettest month (Bio13, 16.6%), slope (4.3%), precipitation of coldest quarter (Bio19, 1.2%), aspect (0.1%), and isothermality (Bio3, 0%). Furthermore, climatic factors collectively accounted for 76% of the total contribution, with precipitation-related variables (39.4) and temperature-related variables (34.6%) dominating. Topographic factors cumulatively contributed 24%. In the Jackknife test (Figure 4b), Bio15, Bio11, and Bio13 were found to yield the most significant increases in regularization training gain when used in isolation, indicating that these variables contain critical ecological information independent of other factors. Following the principle of cumulative contribution ≥ 85% [40,41], Bio11, elevation, Bio15, and Bio13 were established as the dominant environmental driver group, collectively accounting for 94.4% of the contribution and jointly regulating the suitable habitat patterns of switchgrass in China.

**Figure 5:**
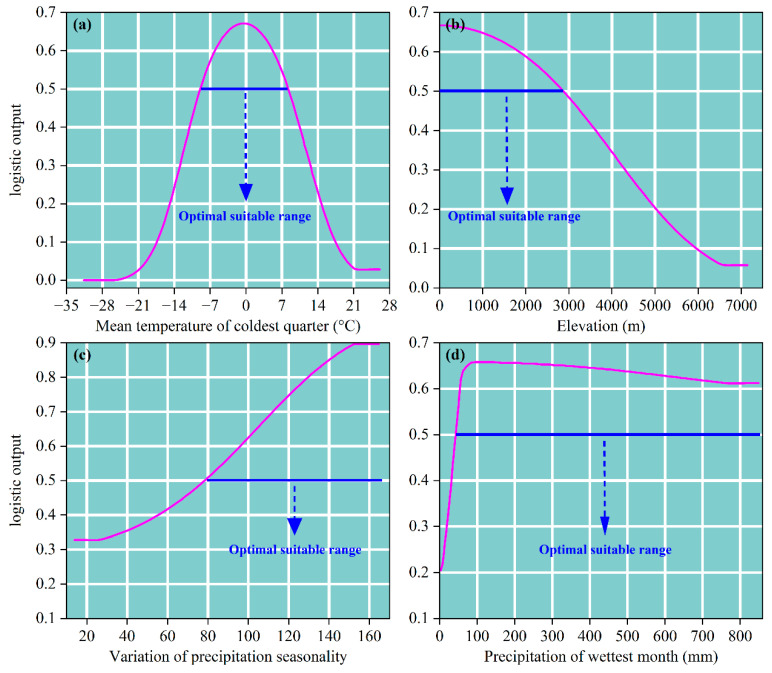
Response curves of dominant environmental factors for switchgrass distribution. (**a**) Response curves of the Bio11; (**b**) response curves of the elevation; (**c**) response curves of the Bio15; (**d**) response curves of the Bio13. Pink curves depict the mean response across 10 replicates, while horizontal solid blue lines denote the optimal suitability range.

The response curves of the dominant environmental drivers (Figure 5) revealed nonlinear patterns in switchgrass habitat suitability probability across key environmental gradients. Bio11 (mean temperature of coldest quarter) was characterized by a unimodal response: suitability probability increased from −24.38 °C to a peak value of 0.67 at −0.32 °C, followed by a sharp decline towards zero at 21.96 °C (Figure 5a). Elevation exhibited a monotonically decreasing pattern: probability remained constant at 0.67 between 0 and 168.96 m, then gradually decayed to zero as elevation increased to 6564.92 m (Figure 5b). Bio15 (variation of precipitation seasonality) displayed a threshold-saturation pattern: suitability probability was maintained at 0.32 when Bio15 < 25.29; it increased rapidly to 0.89 and stabilized within the range of 25.30–152.03 (Figure 5c). Bio13 (precipitation of wettest month) demonstrated an initial increase followed by a gradual decline: probability rose rapidly from 0 mm, reaching a peak of 0.66 at 95.06 mm, then decreased slowly as precipitation increased to 782.80 mm (Figure 5d). Based on the 0.5 probability threshold criterion [42], the optimal suitability thresholds for switchgrass were determined as follows: Bio11, −8.79 to 8.11 °C; elevation, 0 to 2893.09 m; Bio15, 80.12 to 164.98; and Bio13, 42.62 to 848.86 mm.

### 3.3. Current Potential Suitable Habitats of Switchgrass in China

**Figure 6:**
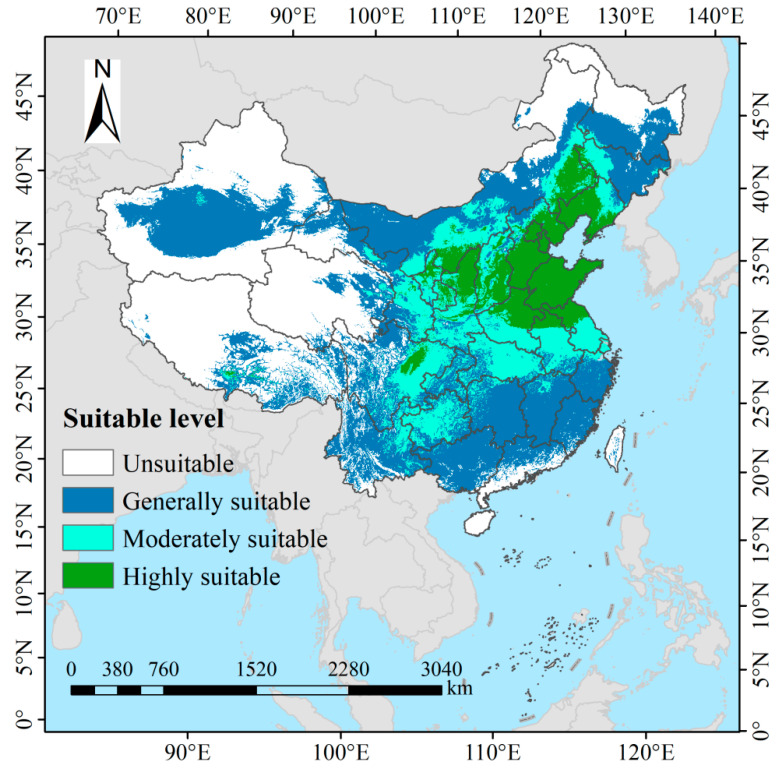
Current potential distribution of switchgrass in China under baseline climate conditions, classified into high (*P* ≥ 0.5), medium (0.3 ≤ *P* < 0.5), low (0.1 ≤ *P* < 0.3), and non-suitable (*P* < 0.1) habitats.

Under current climatic conditions, the total potential suitable habitat area for switchgrass in China is estimated at 583.58 × 10^4^ km^2^, accounting for approximately 60.79% of the country’s total land area (Figure 6). High-suitability habitats (96.21 × 10^4^ km^2^, 10.02%) are centered on the North China Plain, specifically Shandong Province, Hebei Province, Beijing Municipality, Tianjin Municipality, and Liaoning Province, and they extend into eastern Shanxi Province, northern Shaanxi Province, northeastern and southern Inner Mongolia Autonomous Region, northern Henan Province, northern Anhui Province, and northern Jiangsu Province while also spilling over into northeastern Sichuan Province (e.g., Guangyuan, Dazhou, and Bazhong cities). Medium-suitability habitats (145.37 × 10^4^ km^2^, 15.14%) span the middle Yangtze River basin, which embraces Hubei Province, Guizhou Province, and eastern Sichuan Province (e.g., Nanchong and Guang’an cities), together with Chongqing Municipality, and they additionally blanket the southern Loess Plateau, including southern Gansu Province (e.g., Longnan and Tianshui cities) and southern Shaanxi Province (such as Hanzhong, Ankang, and Shangluo cities) while also covering the Jianghuai Basin, illustrated by southern Henan Province (e.g., Xinyang City), southern Anhui Province (e.g., Ma’anshan City), and southern Jiangsu Province (such as Nanjing and Suzhou cities), and further encompassing western Shanxi Province (e.g., Lüliang and Linfen cities) along with western Jilin Province (e.g., Baicheng and Songyuan cities). Low-suitability habitats (342.00 × 10^4^ km^2^, 35.63%) are widely scattered across the southern hill regions that include Chongqing Municipality, Guangxi Zhuang Autonomous Region, Guangdong Province, Yunnan Province, Hunan Province, Jiangxi Province, and Fujian Province, and they also occupy western Sichuan Province (e.g., Garzê Tibetan Autonomous Prefecture and Aba Tibetan and Qiang Autonomous Prefecture), eastern Tibet Autonomous Region (such as Qamdo and Nyingchi cities), central Xinjiang Uygur Autonomous Region (such as Ürümqi city and Changji Hui Autonomous Prefecture), and the northern ecological transition zones, typified by the northern Inner Mongolia Autonomous Region (e.g., Hulunbuir City) and southern Heilongjiang Province (e.g., Harbin and Daqing cities).

### 3.4. Spatiotemporal Patterns of Switchgrass Suitable Habitats Under Future Climate Change Scenarios

**Figure 7:**
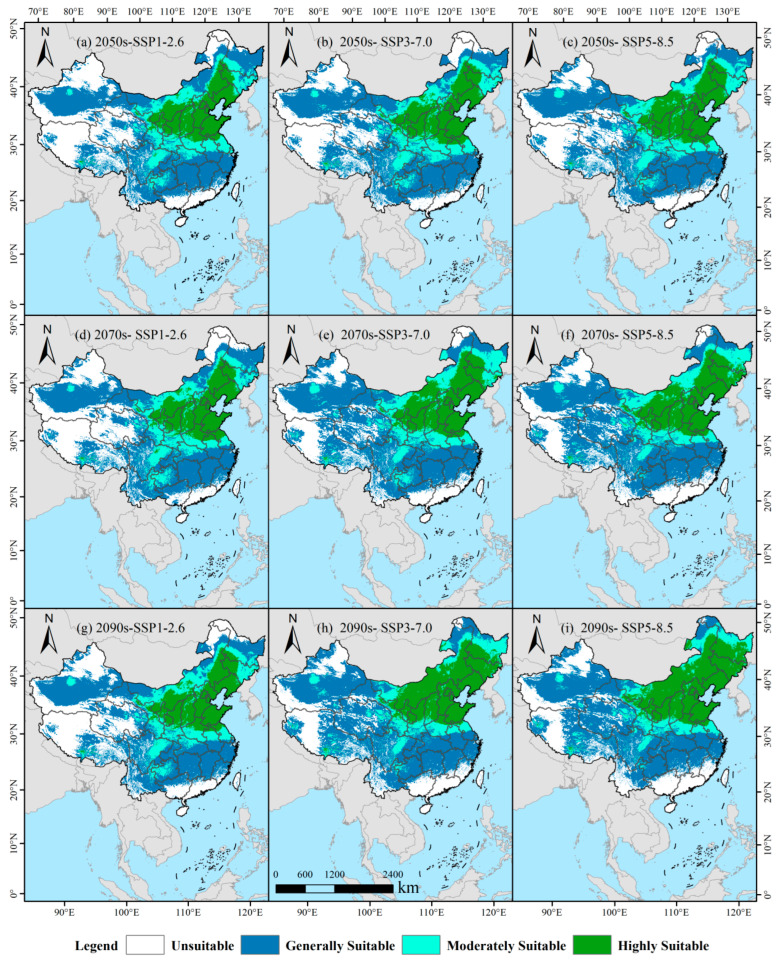
Potential distribution pattern of switchgrass across climate change scenarios. Panels (**a**,**d**,**g**) show the potential distribution of switchgrass under SSP126 across three time periods, while panels (**b**,**e**,**h**) and (**c**,**f**,**i**) display the distributions under SSP3-7.0 and SSP5-8.5 scenarios, respectively.

Under the low-carbon SSP1-2.6 scenario, suitable habitat for switchgrass exhibits a steadily increasing trend (Figure 7a,d,g). By the 2050s, total suitable area is projected at 652.09 × 10^4^ km^2^ (67.93% of China’s land), of which high, medium, and low suitability account for 139.16 × 10^4^ km^2^ (21.3%), 138.38 × 10^4^ km^2^ (21.2%), and 374.54 × 10^4^ km^2^ (57.5%), respectively. High-suitability habitat is concentrated in the North China Plain, covering all of Shandong Province, Hebei Province, Beijing Municipality, Tianjin Municipality, and Liaoning Province and extending into eastern Shanxi Province, northern Shaanxi Province, southern Inner Mongolia Autonomous Region, northern Jiangsu Province, and northern Henan Province. By the 2070s, total suitable area rises modestly to 658.98 × 10^4^ km^2^ (68.64%). High-suitability habitat increases to 146.10 × 10^4^ km^2^ (a 4.9% gain) and now incorporates northern Anhui Province as a contiguous zone, while medium- and low-suitability habitats remain at 138.92 × 10^4^ km^2^ and 373.95 × 10^4^ km^2^, respectively. By the 2090s, total suitable area reaches 663.17 × 10^4^ km^2^ (69.08%). High-suitability habitat expands northward to 153.14 × 10^4^ km^2^ (10% above the 2050s level) and includes southwestern Heilongjiang Province for the first time. Medium-suitability habitat rises slightly to 141.54 × 10^4^ km^2^, whereas low-suitability habitat declines to 368.50 × 10^4^ km^2^. Throughout all three periods, the core North China region (Shandong, Hebei, Beijing, Tianjin, and southern Liaoning) is consistently classified as entirely high suitability.

Under the medium-emission SSP3–7.0 scenario, pronounced spatial reconfiguration of switchgrass habitats is projected (Figure 7b,e,h). By the 2050s, total suitable area reaches 675.79 × 10^4^ km^2^ (70.39% of China’s land), with high-suitability habitat expanding to 150.39 × 10^4^ km^2^, an 8.1% increase relative to SSP1–2.6 for the same period, and a contiguous high-suitability zone already emerges in southwestern Heilongjiang Province. Medium-suitability habitat totals 148.72 × 10^4^ km^2^ (22.0%), and low-suitability habitat covers 376.69 × 10^4^ km^2^. A leapfrog expansion occurs by the 2070s, pushing total suitable area to 734.70 × 10^4^ km^2^ (76.53%). High-suitability habitat grows to 174.13 × 10^4^ km^2^, a 15.8% rise from the 2050s, and extends across the northeastern Inner Mongolia Autonomous Region and western Jilin Province. Medium-suitability habitat remains stable at 150.99 × 10^4^ km^2^, whereas low-suitability habitat surges to 409.58 × 10^4^ km^2^. By the 2090s, total suitable area peaks at 744.86 × 10^4^ km^2^ (77.59%), and high-suitability habitat expands explosively to 209.79 × 10^4^ km^2^, a 39.5% gain over the 2050s, forming a continuous belt centered on northeastern Inner Mongolia and Jilin Province. Concurrently, medium-suitability habitat contracts sharply to 122.48 × 10^4^ km^2^ (a 17.6% reduction), while low-suitability habitat increases slightly to 412.59 × 10^4^ km^2^. Throughout all periods, the core North China region retains uniform high suitability.

Dramatic transformation of the suitable habitat pattern for switchgrass is projected under the high-warming SSP5-8.5 scenario (Figure 7c,f,i). By the 2050s, total suitable area is estimated at 687.09 × 10^4^ km^2^ (71.57% of China’s land), with high-suitability habitats expanding to 167.40 × 10^4^ km^2^ (24.4%), a 20.3% increase compared with SSP1-2.6 for the same period, and emerging hotspots appear in the eastern Inner Mongolia Autonomous Region and southwestern Heilongjiang Province. Medium-suitability habitats occupy 141.75 × 10^4^ km^2^, whereas low-suitability habitats cover 377.93 × 10^4^ km^2^. By the 2070s, total suitable area rises to 734.23 × 10^4^ km^2^ (76.48%), high-suitability habitats reach 186.01 × 10^4^ km^2^ (an 11.1% increase relative to the 2050s), and the entire territory of Jilin Province is incorporated, while medium-suitability habitats contract to 137.58 × 10^4^ km^2^ (a 2.9% decrease) and low-suitability habitats expand to 410.64 × 10^4^ km^2^. By the 2090s, total suitable area peaks at 741.37 × 10^4^ km^2^ (77.22%), high-suitability habitats surge exponentially to 229.44 × 10^4^ km^2^ (a 37.1% increase over the 2050s) and blanket all of eastern Inner Mongolia and central–southern Heilongjiang Province, medium-suitability habitats are substantially reduced to 114.63 × 10^4^ km^2^ (a 21.0% decrease compared with the current baseline), and fragmentation of high-suitability areas is observed in parts of the Huang–Huai Basin, such as southern Henan Province and northern Jiangsu Province. Throughout all periods, the core North China region remains entirely classified as high suitability.

### 3.5. Trends in Suitable Habitat Area Changes for Switchgrass Under Future Climate Scenarios

**Figure 8:**
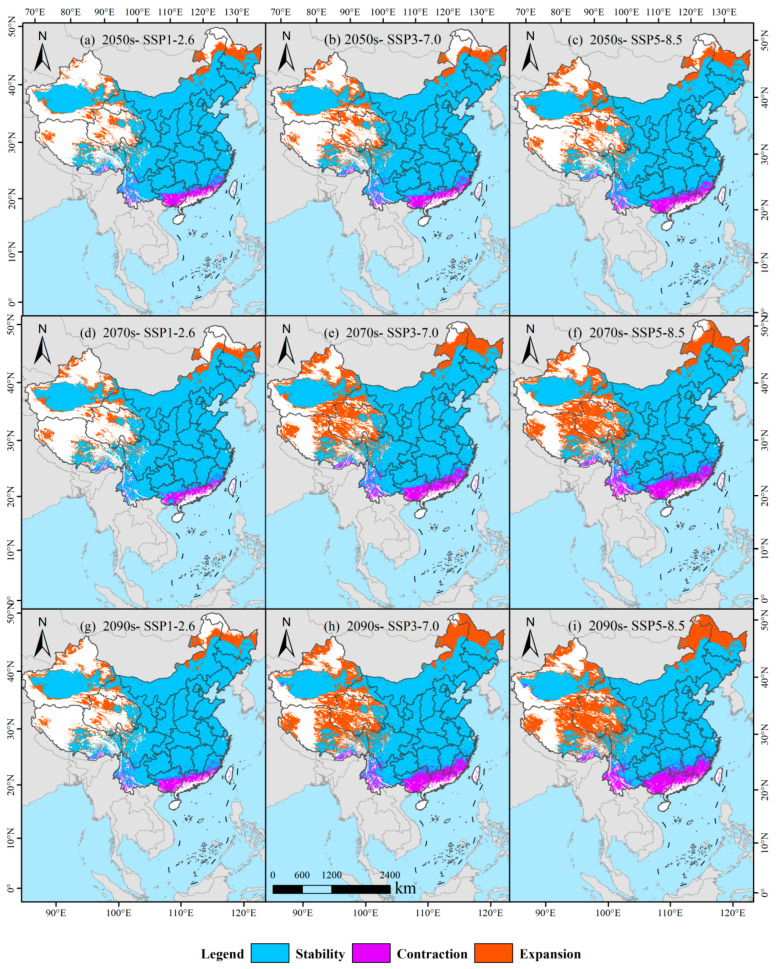
Dynamic changes in the potentially suitable habitat of switchgrass in response to climate change pathways. Panels (**a**,**d**,**g**), (**b**,**e**,**h**), and (**c**,**f**,**i**) depict the dynamic changes in potentially suitable habitats of switchgrass across three time periods under SSP126, SSP3-7.0, and SSP5-8.5 scenarios, respectively.

Nonlinear evolution of suitable habitats for switchgrass occurs under the SSP1-2.6 scenario (Figure 8a,d,g; Table 3). In the 2050s, retained habitat is projected at 681.69 × 10^4^ km^2^ (retention rate: 82.19%), habitat loss zones cover 32.01 × 10^4^ km^2^ (loss rate: 3.86%), and habitat gain zones span 115.66 × 10^4^ km^2^ (gain rate: 13.95%). By the 2070s, the retention rate rise to 88.58% (688.59 × 10^4^ km^2^), the loss rate declines to 3.23% (25.14 × 10^4^ km^2^), and the gain rate contracts to 8.19% (63.65 × 10^4^ km^2^). These trends reverse in the 2090s: the retention rate falls to 80.87% (681.64 × 10^4^ km^2^), the loss rate increases to 3.80% (32.05 × 10^4^ km^2^), and the gain rate expands to 15.33% (129.24 × 10^4^ km^2^). Under this scenario, retained habitats follow a hump-shaped trajectory (681 → 689 → 682 × 10^4^ km^2^), while gain zones exhibit V-shaped fluctuations (116 → 64 → 129 × 10^4^ km^2^), reflecting boundary instability under moderate warming.

Systematic spatial reorganization is triggered by the medium-emission SSP3-7.0 scenario (Figure 8b,e,h; Table 3). In the 2050s, 681.31 × 10^4^ km^2^ is retained (retention rate: 79.34%), 32.41 × 10^4^ km^2^ is lost (loss rate: 3.77%), and 144.98 × 10^4^ km^2^ is gained (gain rate: 16.88%). By the 2070s, the retention rate drops sharply to 71.09% (669.81 × 10^4^ km^2^), the loss rate increases to 4.66% (43.92 × 10^4^ km^2^), and the gain rate rises to 24.25% (228.50 × 10^4^ km^2^). This trend intensifies in the 2090s: the retention rate declines to 68.18% (658.47 × 10^4^ km^2^), the loss rate grows to 5.72% (55.24 × 10^4^ km^2^), and the gain rate exceeds 26.10% (252.12 × 10^4^ km^2^). A unidirectional progression emerges: retained habitats progressively shrink (681 → 670 → 658 × 10^4^ km^2^), loss zones expand linearly (32 → 44 → 55 × 10^4^ km^2^), and gain zones accelerate (145 → 229 → 252 × 10^4^ km^2^), revealing a replacement effect driven by warmer and drier conditions that fragment original core habitats while forming new high-latitude suitability belts.

Dramatic transformation of switchgrass habitats is driven by strong warming under the SSP5-8.5 scenario (Figure 8c,f,i; Table 3). In the 2050s, 674.97 × 10^4^ km^2^ is retained (retention rate: 76.80%), 38.74 × 10^4^ km^2^ is lost (loss rate: 4.41%), and 165.15 × 10^4^ km^2^ is gained (gain rate: 18.79%). By the 2070s, the retention rate declines to 68.47% (654.93 × 10^4^ km^2^), the loss rate increases to 6.14% (58.78 × 10^4^ km^2^), and the gain rate reaches 25.38% (242.78 × 10^4^ km^2^). This pattern persists into the 2090s: the retention rate drops to 66.13% (644.91 × 10^4^ km^2^), the loss rate climbs to 7.05% (68.79 × 10^4^ km^2^), and the gain rate rises to 26.82% (261.53 × 10^4^ km^2^).

### 3.6. Centroid Dynamics of Switchgrass Habitats Across Climate Regimes

**Figure 9:**
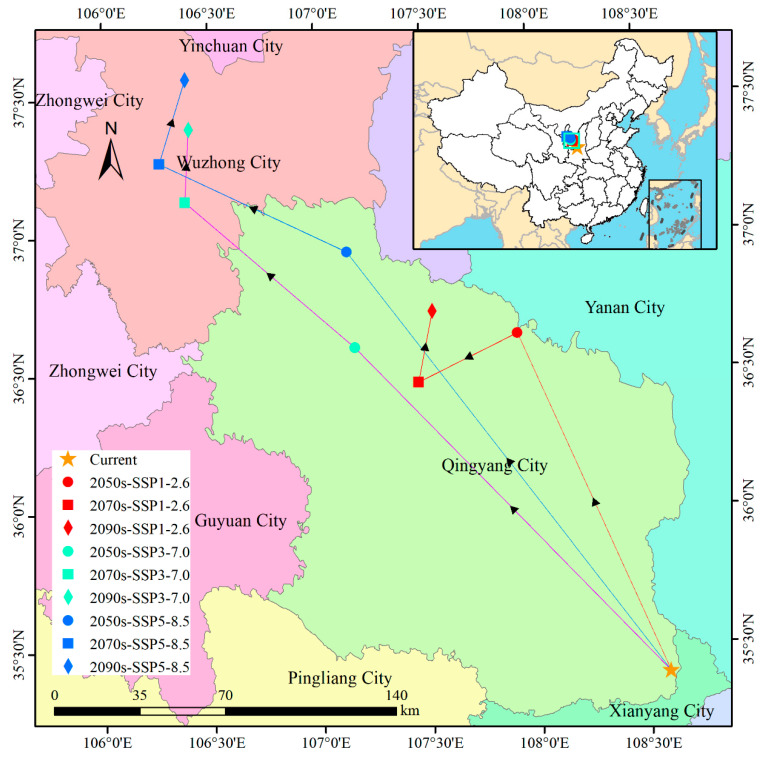
Northwestward centroid migration of switchgrass suitable habitats under SSP scenarios (2050s–2090s). Arrows indicate displacement vectors (km), with terminal points (colored circles, squares, and diamonds) showing final positions. The baseline centroid (orange star) is located in Gansu Province (106.59° E, 35.40° N).

Based on the results derived from ArcGIS 10.8 (Figure 9), the centroid of switchgrass suitable habitats under current climate conditions is located at Wuqingyuan Township, Zhengning County, Gansu Province (108.59° E, 35.40° N). Under SSP1-2.6, the centroid exhibits a tripartite trajectory with defined directional phases. Specifically, by the 2050s, a 150.00 km northwest relocation to Huaian Township, Huachi County, Gansu (107.93° E, 36.64° N) is recorded, corresponding to a mean migration velocity of 7.5 km/yr. Subsequent southwest displacement of 45.68 km to Mubo Town, Huan County, Gansu (107.46° E, 36.47° N) is observed by the 2070s, concurrent with directional realignment and velocity reduction to 2.3 km/yr. By the 2090s, northeast redirection over 29.15 km culminates at Fanjiachuan Town, Huan County (107.53° E, 36.72° N). SSP3-7.0 conditions initiate persistent saltatory displacement northwestward. By the 2050s, the centroid is translocated 184.63 km northwest to Huan Town, Huan County, Gansu (107.17° E, 36.60° N), achieving 9.2 km/yr mean velocity. Acceleration occurs by the 2070s (4.6 km/yr), progressing 91.68 km northwest to Xiamaguan Town, Tongxin County, Ningxia (106.39° E, 37.13° N)—marking provincial boundary transgression. Cumulative northwest movement extends 305.56 km (28° azimuth) by the 2090s via 29.25 km northward relocation to Taiyangshan Town, Hongsibu District (106.41° E, 37.39° N). High-emission SSP5-8.5 scenarios manifest linear, high-magnitude centroid shifts. By the 2050s, 215.61 km northwest displacement (10.8 km/yr) terminates at Qintuanzhuang Township, Huan County, Gansu (107.14° E, 36.94° N). Continuation northwestward for 85.58 km by the 2070s reaches Xinzhuangji Township, Hongsibu District, Ningxia (106.27° E, 37.27° N), involving a 0.86° longitudinal westerly shift (~73 km). The final 35.53 km northeast translocation by the 2090s positions the centroid at Taiyangshan Town (106.39° E, 37.58° N).

## 4. Discussion

### 4.1. MaxEnt Model Optimization and Performance Evaluation for Switchgrass

Overfitting risk was significantly reduced through optimized MaxEnt parameters (RM = 4.0, FC = LQH), as demonstrated by Delta.AICc = 0 and OR10 = 0.125 versus the default 0.146. Traditional MaxEnt applications relying on default settings (e.g., RM = 1.0, FC = LQHP) are widely recognized as overestimating species’ climatic tolerance, particularly in topographically complex regions [32,34]. For instance, uncalibrated models underestimate cold-season constraints, resulting in overestimation of suitable habitats for high-latitude C4 grasses [21,22]. The ENMeval 2.0.4 toolkit was employed to systematically test 128 parameter combinations, thereby validating the finding of Kass et al. [32] that customized RM/FC configurations enhance ecological niche realism. Model robustness was confirmed by an AUC (area under the receiver operating characteristic curve) value of 0.856 ± 0.069, exceeding the 0.8 “high predictive accuracy” threshold established by Swets [39] and Phillips et al. [34]. Limitations inherent to modeling the distribution of switchgrass were addressed through stringent spatial filtering (48 independent occurrence points) and multicollinearity control (|r| < 0.75). Potential overestimation of suitable areas on the Loess Plateau, as indicated by Wang et al. [17], was attributed to spatial autocorrelation caused by clustered experimental plot data in earlier Chinese studies [21]. A composite habitat framework integrating topographic factors (elevation and slope) and climatic variables was developed, substantiating the core theory proposed by Liu et al. [35] that terrain–climate interactions are fundamental drivers of species distribution model accuracy.

### 4.2. Dominant Environmental Factors Governing Switchgrass Habitat Suitability

Habitat suitability for switchgrass in China was primarily determined by the mean temperature of the coldest quarter (Bio11, contribution rate 39.4%) and elevation (21.6%), reflecting the species’ sensitivity to freezing damage and hypoxia. The optimal Bio11 range (−8.79 to 8.11 °C) corresponded to ecophysiological theory in which rhizome injury occurred below −10 °C and dormancy cycles were disrupted above 15 °C [5,6]. This pattern explains the current concentration of highly suitable areas in the North China Plain (Figure 6), where Bio11 averages −2 to 5 °C. Suitability declined monotonically with increasing elevation, peaking at 0–2893 m, a trend confirmed by field trials in karst mountain regions; cultivation efficiency was 30–50% higher in lowlands because of superior thermal conditions [8,19]. Isothermality (Bio3) contributed negligibly (≈0%), a result that contrasts sharply with the findings of Ahrens et al. [20], who identified it as a key factor in North America. This discrepancy underscores the phenotypic plasticity that has developed during the species’ domestication in China [17,18].

Medium-to-low suitability zones for switchgrass were jointly shaped by precipitation seasonality (Bio15, 16.8%) and precipitation of the wettest month (Bio13, 16.6%). A threshold response in Bio15 was identified, with habitat suitability probability sharply increasing above 25.29, indicating the species’ dependence on stable rainy-season moisture for establishment, consistent with irrigation experiments in northwestern China [7]. A unimodal response curve for Bio13 (optimum 95.06 mm) revealed vulnerability to extreme rainfall, as waterlogging-induced suppression of foliar photosynthesis and transpiration reduced aboveground biomass accumulation and indirectly diminished root biomass by 20–40%, particularly in micro-topographic depressions prone to ponding [43]. Systematic overestimation of suitability in the Yangtze River Basin by earlier studies [21,44] was attributed to the direct use of uncorrected WorldClim bioclimatic variables (e.g., Bio12 annual precipitation) while neglecting critical thresholds of precipitation seasonality (Bio15) and extreme rainfall (Bio13). The marginal role of precipitation of the driest quarter (Bio19, 1.2%) contrasted with the findings of [5], which emphasized drought tolerance in U.S. ecotypes, and suggests potential cold-hardiness trade-offs at the expense of drought resistance in Chinese germplasm, a strategic balance proposed for optimization through breeding gene selection [6].

### 4.3. Projected Shifts in Potential Switchgrass Distribution Under Climate Change

Under the SSP5-8.5 scenario, high-suitability areas for switchgrass are projected to expand by 138 percent, reaching 229.44 × 10^4^ km^2^ by the 2090s, while the distribution centroid is expected to shift 305 km northwestward (Figure 9). This redistribution is attributed to climatic adaptation mechanisms embedded in the species’ genome: thermal constraints at high latitudes are alleviated through introgression of cold-tolerance alleles, and adaptive evolution is accelerated by subgenome differentiation in polyploid lineages. Concurrently, suitability declines in southern ecotypes are driven by respiratory carbon loss under elevated temperatures [5]. The North China Plain remains a perennial high-suitability zone across all SSP scenarios, as the projected increase in Bio11 (≈4.5 °C by the 2090s) stays within the species’ optimal growth range [11]. However, the dramatic expansion projected for Inner Mongolia (SSP5-8.5: increasing 261.53 × 10^4^ km^2^ by the 2090s) should be interpreted cautiously because sandy soils may limit biomass accumulation [45,46]. Crucially, 7.05 percent of habitat loss is concentrated in southwestern China under SSP5-8.5, where warming surpasses the upper thermal threshold of the optimal Bio11 range (8.11 °C), supporting the findings of Wang et al. [47] on heat-stress-induced yield reductions in the Yunnan–Guizhou Plateau.

Although switchgrass possesses inherent drought tolerance as a C4 species [5], our results demonstrate climate-driven habitat redistribution rather than uniform expansion across China. While warming facilitates significant northward range extension (+138% area gain under SSP5-8.5), approximately 7% of current southern habitats become unsuitable due to exceeding optimal thermal thresholds (Bio11 >8.1 °C; Figure 7). This spatial dichotomy reflects three key factors: (1) latitudinal compensation, where cold limitation is alleviated in northern regions while heat stress intensifies in the south; (2) regional adaptation limits, as Chinese cultivars exhibit narrower thermal tolerance compared to North American ecotypes [21]; and (3) non-climatic constraints, particularly soil-texture barriers in arid expansion zones [45]. Consequently, strategic cultivation planning must account for these opposing regional trends, emphasizing development of cold-tolerant varieties for newly suitable northern areas while maintaining southern germplasm banks to preserve genetic diversity. This balanced approach ensures sustainable switchgrass utilization under China’s varying climate change impacts.

A consistent northwestward shift in the distribution centroid of switchgrass suitability was identified across three climate scenarios in our study, indicating that arid zones could function as future climatic refugia. This finding is consistent with Pau et al. [4], where analogous migrations were documented in thermophilic C4 herbs. Consequently, we recommend integrating migration dynamics with China’s Marginal Land Bioenergy Strategy [48,49,50,51] through the following measures: (1) designation of priority cultivation belts in northwestern arid regions under energy crop policies; (2) promotion of drought-tolerant cultivars supported by subsidy frameworks; and (3) formulation of BECCS industrial coupling mechanisms [52] to incentivize integrated carbon-negative supply chains. Nevertheless, under SSP3-7.0/SSP5-8.5 scenarios, rapid habitat expansion (26.1–26.8% increase) is projected to exceed human-assisted migration capacity, forcing accelerated transplantation under conditions in which inadequate screening of edaphic and biotic conditions is predicted to induce maladaptation through survival rate decline [53]. Concurrently, it is recognized that genetic connectivity in the Yellow–Huai Basin is threatened by intensifying landscape fragmentation [54,55], necessitating preemptive ecological corridors [56,57]. To address climate-forced northward migration of herbaceous flora [58,59,60], cold-tolerant hybrids via interspecific hybridization [61,62,63] are advocated to buffer extinction risks while enabling carbon-negative systems through dual mechanisms: photosynthetic sequestration on idle lands and fossil fuel displacement via combustion [64,65].

### 4.4. Limitations and Future Research Directions

Significant advances were achieved in MaxEnt parameter optimization, scenario coupling, and centroid migration quantification within this study. However, three principal limitations were identified that constrain its transition from “suitability simulation” to “policy implementation” in terms of precision and credibility. First, critical stress factors were not incorporated into the model, including soil texture, salinization, extreme low-temperature event frequency, and differential CO_2_ fertilization effects [66,67]. These variables exert nonlinear, amplified influences on rhizome survival, photosynthetic efficiency, and carbon sequestration function in marginal lands and arid zones. Simultaneously, the absence of dynamically coupled pest/pathogen northward expansion and interspecific competition mechanisms may lead to underestimation of actual productivity in high-latitude expansion zones. Second, genotype–environment feedbacks were undifferentiated; treating all Chinese ecotypes as a single unit overlooked ploidy-level trade-offs (e.g., 4× vs. 8× cold/drought tolerance). Future studies should integrate cytotype-specific genetic data to stratify niche modeling, enhancing realism for regional deployment. Consequently, projections of future gene-flow directions and local adaptation rates may be overly optimistic. Third, policy–climate synergistic scenarios were lacking. Current SSP frameworks failed to integrate socioeconomic levers such as land-use conversion rates under the Dual-Carbon Goals, water resource quotas, and biomass procurement pricing [68,69,70]. This omission precludes evaluation of anthropogenic acceleration or blocking effects on habitat area and migration trajectories resulting from policy interventions. Fourth, while our model effectively captures climate–topography interactions, it does not incorporate three critical anthropogenic dimensions that may influence real-world distributions: (1) dynamic land-use conversion rates under China’s evolving ecological policies, (2) practical cultivation constraints in urbanized/agricultural regions, and (3) potential CO_2_ fertilization effects on C_4_ photosynthesis. Fifth, our exclusive focus on biophysical variables represents a key limitation, as socioeconomic pathways inherently embed assumptions about land-use governance, grassland management intensity, and conservation investment that directly impact species distributions. The divergence between SSP storylines, from SSP126’s coordinated sustainability to SSP585’s fossil-fueled development, implies vastly different policy landscapes that could override climate suitability patterns.

Future efforts should establish a four-dimensional “climate–soil–genome–policy” coupling framework as follows: (1) Phenotypic plasticity thresholds of distinct cytotypes under interannual climate extremes should be quantified via common garden experiments and genome–environment association analyses, enabling derivation of genotype-specific response functions. (2) Correlative statistical models should be replaced by process-based mechanistic growth–mortality models that integrate CMIP6-derived high-resolution indicators (soil moisture, heatwaves, and cold spells) with marginal-land soil databases. (3) An agent-based land-use/market response module should be constructed to dynamically project spatial pattern feedbacks from farmer decisions under carbon pricing, subsidy, and water allocation scenarios. (4) Future modeling efforts should adopt an integrated framework that couples habitat suitability projections with high-resolution land-change datasets, incorporates marginal land quality inventories, and evaluates genotype-specific CO_2_ response curves through controlled experiments. Ultimately, an interactive digital twin platform for switchgrass suitability should be developed around the integrated genome–environment–policy model to deliver actionable decision support for regional germplasm deployment and climate-smart bioenergy corridor planning.

## 5. Conclusions

This study, based on 48 spatially independent occurrence records, systematically delineated the suitable habitat of switchgrass in China and its spatiotemporal evolution from the 2050s to 2090s for the first time. BCC-CSM2-MR climate scenarios were coupled with topographic variables, and MaxEnt was optimized (RM = 4.0, FC = LQH) using ENMeval. Results indicate that the current suitable habitat covers 60.8% of China’s land area, with the core high-suitability zone concentrated in the North China Plain. Bio11 (mean temperature of the coldest quarter) and elevation collectively contribute 61.0%, making them the primary limiting factors. Under all three SSPs, habitat area is projected to expand; by the 2090s under SSP5-8.5, the high-suitability area is expected to reach 2.29 × 10^4^ km^2^, and the habitat centroid is expected to shift 305 km northwest. Inner Mongolia–Jilin is projected to emerge as the new primary production region, whereas 7% habitat loss is anticipated in the southwest due to excessive heat. The climate–topography coupling mechanism driving northward migration of switchgrass is thus revealed. Future efforts are recommended to focus on breeding cold-tolerant cultivars, prioritizing the delineation of marginal land zones, and establishing ecological corridors. These strategies are proposed as critical breakthroughs to support the planning of a climate-smart biomass energy belt in China under the dual-carbon goals.

## Figures and Tables

**Table 1:** Environmental factors employed to project the suitable habitats of switchgrass in China.

Variables	Description	Units	Range	Contribution Rate (%)
Bio1	Annual mean temperature	°C	2.64–21.16	4.1
Bio2	Mean diurnal range	°C	6.40–14.42	1.3
**Bio3**	**Isothermality**		**22.37–50.71**	**0.4**
Bio4	Standard deviation of temperature seasonality		351.21–1523.36	0.3
Bio5	Max temperature of warmest month	°C	15.48–33.58	4.1
Bio6	Min temperature of coldest month	°C	−24.53–8.87	0.1
Bio7	Temperature annual range	°C	21.43–52.60	0.3
Bio8	Mean temperature of wettest quarter	°C	9.62–26.56	0.2
Bio9	Mean temperature of driest quarter	°C	−15.93–17.78	1.3
Bio10	Mean temperature of warmest quarter	°C	9.62–28.23	2.4
**Bio11**	**Mean temperature of coldest quarter**	**°C**	**−15.93–16.18**	**18.2**
Bio12	Annual precipitation	mm	174.00–1601.00	0
**Bio13**	**Precipitation of wettest month**	**mm**	**50.00–316.00**	**18.1**
Bio14	Precipitation of driest month	mm	0.00–45.00	0.2
**Bio15**	**Variation of precipitation seasonality**		**50.32–148.55**	**19.9**
Bio16	Precipitation of wettest quarter	mm	112.00–837.00	0
Bio17	Precipitation of driest quarter	mm	2.00–180.00	0
Bio18	Precipitation of warmest quarter	mm	107.00–829.00	0.1
**Bio19**	**Precipitation of coldest quarter**	**mm**	**2.00–201.00**	**10.6**
**Elevation**	**Elevation**	**m**	**1.00–4178.00**	**9.8**
**Aspect**	**Aspect**		**17.35–332.40**	**3.6**
**Slope**	**Slope**	**°**	**0.00–3.10**	**4.8**

Note: The eight variables ultimately retained in the final model are highlighted in bold.

**Table 2:** Model performance metrics for MaxEnt across parameter configurations.

Model Type	Feature Combination	Regularization Multiplier	Delta.AICc	OR10	AUC.diff
Default model	LQHP	1	31.229	0.146	0.119
Optimize model	LQH	4	0	0.125	0.110

**Table 3:** Projected gains and losses of switchgrass habitats across climate scenarios.

Period	Area (10^4^ km^2^)			Rate of Change (%)		
	Stability	Contraction	Expansion	Stability	Contraction	Expansion
2050s-SSP1-2.6	681.69	32.01	115.66	82.19	3.86	13.95
2070s-SSP1-2.6	688.59	25.14	63.65	88.58	3.23	8.19
2090s-SSP1-2.6	681.64	32.05	129.24	80.87	3.80	15.33
2050s-SSP3-7.0	681.31	32.41	144.98	79.34	3.77	16.88
2070s-SSP3-7.0	669.81	43.92	228.50	71.09	4.66	24.25
2090s-SSP3-7.0	658.47	55.24	252.12	68.18	5.72	26.10
2050s-SSP5-8.5	674.97	38.74	165.15	76.80	4.41	18.79
2070s-SSP5-8.5	654.93	58.78	242.78	68.47	6.14	25.38
2090s-SSP5-8.5	644.91	68.79	261.53	66.13	7.05	26.82

## Data Availability

The original contributions presented in this study are included in this article. Further inquiries can be directed to the corresponding author.
